# Granulomatous Peritonitis Secondary to Primary Sclerosing Cholangitis

**DOI:** 10.7759/cureus.34738

**Published:** 2023-02-07

**Authors:** Ioannis Ketsekioulafis, Evanthia Serpetsidaki, Georgios Tribonias, Antonios Vezakis, Despoina Myoteri

**Affiliations:** 1 Department of Pathology, National and Kapodistrian University of Athens, School of Medicine, Aretaieion University Hospital, Athens, GRC; 2 Department of Pathology, Venizeleio General Hospital, Heraklion, GRC; 3 Department of Gastroenterology, General Hospital Nikaia Piraeus Agios Panteleimon-General Hospital Dytikis Attikis Agia Varvara, Athens, GRC; 4 Department of Surgery, National and Kapodistrian University of Athens, School of Medicine, Aretaieion University Hospital, Athens, GRC

**Keywords:** liver pathology, peritoneal disease, peritoneal carcinomatosis, primary sclerosing cholangitis, granulomatous peritonitis

## Abstract

Primary sclerosing cholangitis (PSC) is a rare, chronic, and progressive disease of the liver characterized by cholestasis due to multifocal bile duct strictures. PSC can lead to liver fibrosis, and in 10-20% of cases, it leads to cholangiocarcinoma and end-stage liver disease. However, the pathogenesis of the disease is not clearly understood. For the diagnosis of PSC, both imaging and liver biopsy can be used. No medical treatment has managed to prevent the progression of the disease. Consequently, in the case of late-stage disease, liver transplantation is considered the best treatment option. PSC may lead to different complications including bacterial cholangitis, cholangiocarcinoma, and cirrhosis. Nevertheless, to our knowledge, there are no reports of granulomatous peritonitis secondary to PSC. Granulomatous peritonitis may be a result of infectious, malignant, and idiopathic inflammatory diseases. It is also considered a rare postoperative complication, due to cornstarch from surgical glove powder, in laparoscopic procedures. Here, we report the case of a 39-year-old male patient with PSC, in which cholangiocarcinoma and peritoneal carcinomatosis were clinically suspected. Despite that, histological findings and staining methods of the surgically removed peritoneal masses demonstrated granulomatous peritonitis.

## Introduction

Primary sclerosing cholangitis (PSC) is a rare, chronic, and progressive liver disease identified by multifocal bile duct strictures which result in cholestasis [1-3]. The incidence of the disease is 0-1.3 per 100,000 population/year and the prevalence is up to 16 per 100,000 population. PSC is most often diagnosed in men compared to women and between the ages of 30 and 40 years [2]. It can also co-exist with inflammatory bowel disease (IBD), leading to colorectal neoplasia. The disease complications include bacterial cholangitis, dominant strictures, cholangiocarcinoma, cirrhosis, and osteoporosis [1,3]. However, to our knowledge, there are no reports of PSC complicated by granulomatous peritonitis. Granulomatous peritonitis is characterized by a granulomatous reaction that can be histologically diagnosed and can occur due to infectious, malignant, and idiopathic inflammatory diseases, such as IBD. It is also considered to be a rare postoperative complication because of a delayed cell-mediated response to cornstarch from surgical glove powder in open procedures and less commonly in laparoscopic procedures [4,5].

## Case presentation

We report a case of PSC mimicking cholangiocarcinoma and peritoneal carcinomatosis. A 39-year-old male with a history of PSC and extensive ulcerative colitis (UC) (Montreal Classification: E3) visited our department with recurrent episodes of biliary colic in the previous three months. The patient had been followed up annually for longstanding PSC (14 years) by magnetic resonance cholangiopancreatography (MRCP) and cancer antigen 19-9 blood test. The last MRCP demonstrated the previously known and documented intra and extrahepatic strictures without any differentiation. The only new findings highlighted were choledocholithiasis with prestenotic dilation of the common bile duct (CBD) above the dominant stricture in the middle of the CBD and concurrent cholelithiasis.

The patient underwent endoscopic retrograde cholangiopancreatography (ERCP) with stone extraction, brushing for cytology of the dominant stricture, balloon dilatation, and stent placement electively. The biliary cytology revealed a strong suspicion for malignancy, specimen (class IV), and the patient was rescheduled for ERCP and simultaneous cholangioscopy (SpyScope DS, Boston Scientific). The biopsy sample of the stricture was indefinite for dysplasia.

**Figure 1:**
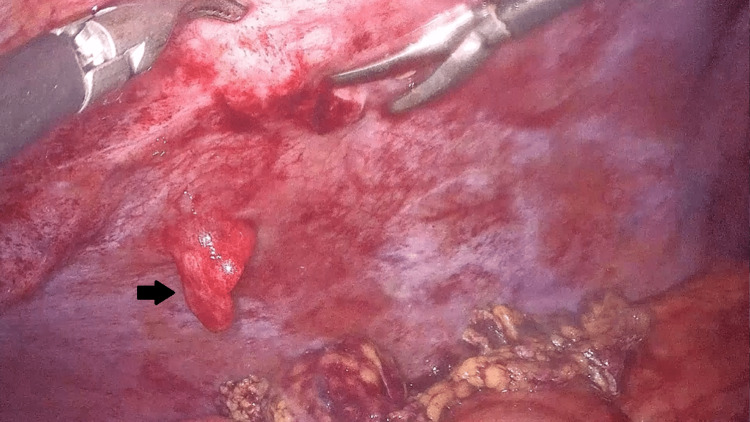
Detected from CT abdominal mass on diagnostic laparoscopy. A 3-cm right-sided abdominal mass with a high suspicion of intraperitoneal seeding arising from an intrahepatic cholangiocarcinoma.

One month later, the patient presented with fever, chills, and elevated serum bilirubin in blood lab work. It was decided to proceed with laparoscopic cholecystectomy because of the gallbladder hydrops and cholelithiasis. Even though he had a transient amelioration of his symptoms, in the following days, he had a high fever as well as severe right lower quadrant pain and abdominal distention with hydrocele. An emergency CT scan demonstrated ascites and a 3-cm right-sided abdominal mass with a high suspicion of intraperitoneal seeding arising from an intrahepatic cholangiocarcinoma (Figure 1).

**Figure 2:**
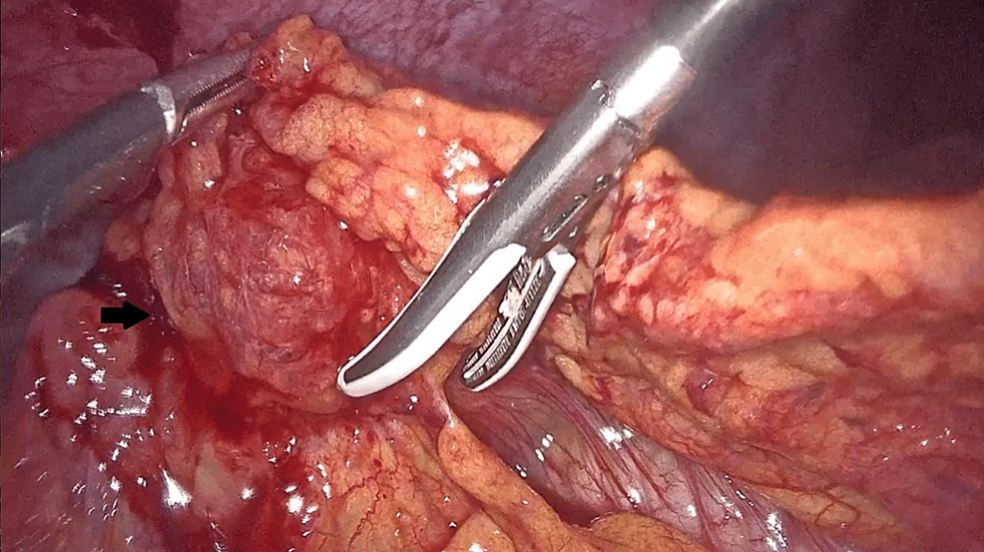
Nodule adherent to the abdominal wall on diagnostic laparoscopy. A 1.5-cm abdominal wall nodule adherent to the anterior abdominal wall.

A diagnostic laparoscopy was performed with ascitic fluid analysis and culture. The detected mass as well as a 1.5-cm abdominal wall nodule adherent to the anterior abdominal wall were removed (Figure 2).

**Figure 3:**
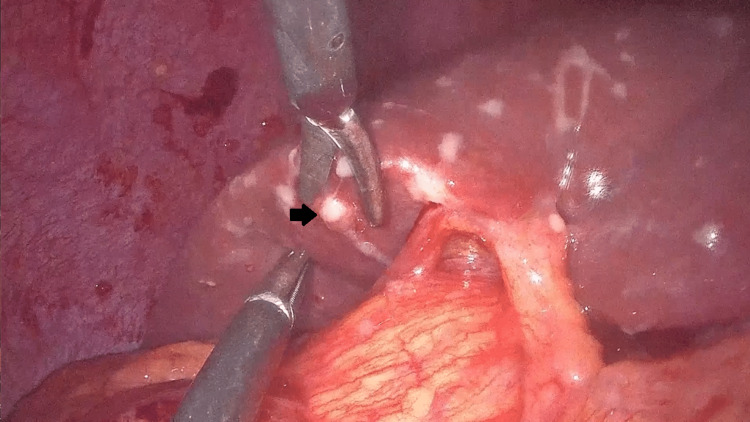
Whitish nodules on the liver resembling peritoneal carcinomatosis. Widespread detection of multiple whitish nodules in the peritoneum.

Based on the laparoscopic images of the masses and the widespread detection of multiple whitish nodules in the peritoneum, the diagnosis of peritoneal carcinomatosis was set with reasonable certainty (Figure 3).

**Figure 4:**
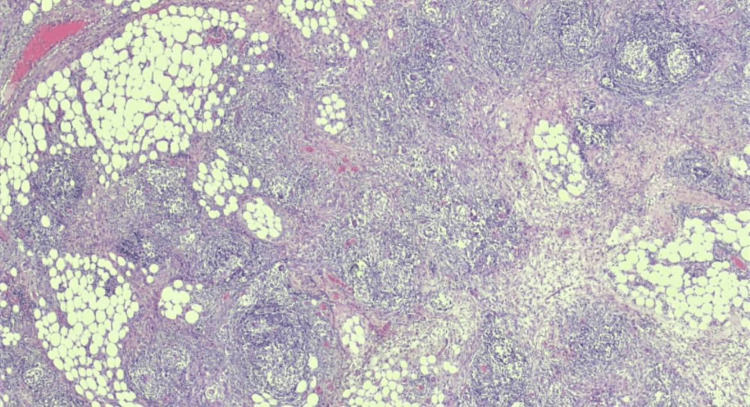
Granulomas on hematoxylin and eosin staining (×20 magnification). Hematoxylin and eosin stain (×20 magnification) demonstrating granulomatous peritonitis.

However, peritoneal carcinomatosis was later excluded histopathologically. After the tissue sampling, granulomas were demonstrated. Histological findings and staining methods of the surgically removed masses were negative for tuberculosis, sarcoidosis, fungal infections and IgG4-related sclerosing mesenteritis, and other granulomatous diseases, confirming a diagnosis of granulomatous peritonitis (Figure 4).

## Discussion

PSC is a rare, chronic, and progressive liver disease marked by cholestasis because of multifocal bile duct strictures that affect both intra and extraepithelial bile ducts. Genetic factors are considered to concur about 10% of the predisposition for the disease, highlighting that PSC is likely immune-mediated and triggered by human leukocyte antigen-restricted T cells leading to the release of profibrogenic cytokines [1-3]. However, the etiology and pathogenesis of the disease are not clearly understood [1].

Patients with PSC may remain asymptomatic for a long period of time. However, some symptoms may occur even in the early stages of the disease. These symptoms include pruritus, fatigue, right upper quadrant abdominal pain, and fever and are considered to be signs of disease complications. The complications of PSC include bacterial cholangitis, dominant strictures, cholangiocarcinoma, cirrhosis, and osteoporosis [1-3]. Among patients with PSC, 10-20% will develop cholangiocarcinoma. This fact results in the consideration of PSC as a premalignant disease [2].

The management of patients with symptoms that are suspected of PSC may begin with serum markers as patients with PSC often show elevated levels of markers of cholestasis such as alkaline phosphatase, γ-glutamyl transferase, and conjugated bilirubin, especially when PSC is combined with IBD. Autoantibodies may also help in the diagnosis of PSC as negative results of antimitochondrial antibodies and primary biliary cirrhosis-specific antinuclear antibodies will lead to the exclusion of primary biliary cholangitis.

Imaging techniques play a pivotal role in the diagnosis of PSC. Ultrasound can help in the exclusion of other diseases, such as some causes of secondary sclerosing cholangitis, and magnetic resonance cholangiography is considered the primary diagnostic imaging modality in patients with suspected PSC. Liver biopsy is considered irreplaceable in some cases, even though it is not routinely required due to invasiveness and risk of complications. Specifically, liver biopsy leads to the determination of the stage of PSC, providing clues regarding the disease progression, long-term outcomes, and transplantation-free survival rates. In general, the classic histological hallmark of PSC is the so-called onion-skin periductal fibrosis. However, this feature is often not found in biopsy specimens, particularly in early-stage disease.

Despite extensive research, no medical treatment has managed to prevent disease progression. As a result, liver transplantation remains the best treatment option for late-stage disease [1-3].

On the other hand, granulomatous peritonitis is a rare disease characterized by a granulomatous reaction that can be histologically diagnosed. It occurs due to infectious, malignant, and idiopathic inflammatory diseases, such as IBD. It is also considered a rare postoperative complication caused by a delayed cell-mediated response to cornstarch from surgical glove powder in open procedures and less commonly in laparoscopic procedures. Previous studies have reported the different causes of granulomatous peritonitis, such as ascariasis, iatrogenic spillage, and starch [4-7].

We presented the case of a patient with PSC and UC who developed granulomatous peritonitis. Even if it is not possible to demonstrate the exact pathophysiology of this patient’s granulomatous peritonitis, different scenarios can be presented. First, it has to be mentioned that one possible scenario that led this patient to develop granulomatous peritonitis could be bile leakage into the peritoneum as it is considered a foreign body in relation to the peritoneal space. However, this was not recorded in this patient as the gallbladder was intact and no leakage from the biliary tree was reported. On the other hand, the fact that the patient underwent ERCP and laparoscopic surgery may have led to granulomatous peritonitis can also be considered a risk factor. Another possible scenario might be that granulomatous peritonitis was a complication of the patient’s known UC as it is an idiopathic inflammatory disease that is connected with this kind of peritonitis. However, there are no cases reporting granulomatous peritonitis secondary to PSC, which is also an idiopathic inflammatory disease and might have played a pivotal role in the development of granulomatous peritonitis in this patient.

## Conclusions

PSC is a progressive disease of the liver that presents with cholestasis, while granulomatous peritonitis is a disease caused by infection, malignancy, idiopathic inflammation, and, rarely, delayed cell-mediated response to cornstarch from surgical glove powder. Both PSC and granulomatous peritonitis rarely occur individually, with no concurrent detection reported yet in the literature. We presented the case of a patient with PSC who developed granulomatous peritonitis which mimicked peritoneal carcinomatosis both on imaging and intraoperatively. The possibility of the pathogenesis of granulomatous peritonitis secondary to PSC needs to be further investigated.
